# Species-specific physiological status in seabirds: insights from integrating oxidative stress measurements and biologging

**DOI:** 10.3389/fphys.2025.1509511

**Published:** 2025-03-19

**Authors:** Shiho Koyama, Yuichi Mizutani, Yusuke Goto, Ken Yoda

**Affiliations:** Graduate School of Environmental Studies, Nagoya University, Nagoya, Japan

**Keywords:** marine animals, antioxidants, pro-oxidant, gulls, shearwaters, BAP, d-ROMs, GPS

## Abstract

Understanding the relationship between behavior and physiological state, as well as species differences in physiological responses, is key to identifying the behavioral and physiological adaptations necessary for wild animals to avoid physiological deterioration, thereby enhancing their survival and fitness. A commonly used measure of physiological condition is oxidative stress, which results from an imbalance between oxidative damage—often exacerbated by respiration during exercise and indicative of physical harm—and antioxidant capacity, which reflects the organism’s ability to recover from such damage. Despite its importance, oxidative stress has rarely been linked to behavior, such as foraging, leaving this relationship underexplored. In this study, we focused on two seabird species, black-tailed gulls (*Larus crassirostris*) and streaked shearwaters (*Calonectris leucomelas*), which are similar in body size and primarily forage on the same prey species but differ in traits such as habitat, flight style, and physiological function. We recorded the trajectories of these birds for approximately 1 week using biologging and measured their plasma oxidative stress. We found that oxidative stress in black-tailed gulls was higher than that in streaked shearwaters, suggesting that species differences in life histories, habitats, and physiological function may be related to long-term oxidative stress. However, over a 1-week timescale, there were no significant species differences in changes in oxidative stress, suggesting that behavioral differences between the two species might not necessarily lead to species-specific oxidative stress responses in the short term. Additionally, no consistent relationship was found between changes in oxidative stress of the two species and their behavioral metrics in most years, suggesting that this relationship may vary depending on yearly environmental fluctuations. Based on our findings, we encourage future studies that would explore and integrate the interactions between marine environments, behavior, and oxidative stress of different bird species to clarify the contribution of specific foraging behaviors to either the deterioration or recovery of physiological conditions, and the varying effect of environmental conditions on these relationships.

## 1 Introduction

Wild animals attempt to minimize physiological deterioration, such as reduction in nutrition (Bishop et al., 2009) and immunity (Cabezas et al., 2011) and increase in stress hormones (Kitaysky et al., 2007; Romero and Wikelski, 2010; Wey et al., 2015) and oxidative stress (Noguera et al., 2012; Costantini and Dell’Omo, 2015), that can reduce their survival and fitness. In particular, long-lived animals with multiple breeding opportunities are likely to behave efficiently to minimize physiological deterioration and successfully raise offspring during the breeding period to ensure both current and future reproductive success by adjusting their foraging and provisioning behaviors, such as locomotion style, time spent foraging, and the distance to the foraging site (Killen et al., 2013; Cooke et al., 2014; Crino et al., 2017). These behavioral adaptations can vary significantly among species based on their life history and habitat, leading to differences in how foraging and provisioning behaviors impact physiological conditions across species. To understand species-specific behavioral adaptation, it is necessary to assess which foraging and provisioning behaviors contribute to either the deterioration or recovery of physiological conditions and how these differ between species.

In addition to individual-level responses, wild animals are expected to develop species-specific tolerance to physiological deterioration depending on their habitat and life history (McCue, 2010; Bozinovic et al., 2011). Laboratory experiments have demonstrated species differences in physiological conditions and survival under stressors such as starvation, drugs, and parasites (Haley, 2003; Matzkin et al., 2009; Rohr et al., 2010). Evaluating species differences in tolerance of wild animals may lead to a deeper understanding of their physiological adaptations to habitats and life histories (Hermes-Lima and Zenteno-Savín, 2002; Kroeger et al., 2019).

Oxidative stress, an indicator of physiological condition, results from an imbalance between oxidative damage, such as reactive oxygen species (ROS) generated by respiration, and antioxidant capacity (Sies, 1997; Urso and Clarkson, 2003). While ROS can eliminate pathogens in body tissues, excessive accumulation damages biomolecules such as DNA, proteins, and lipids, leading to physiological deterioration (Filomeni et al., 2015; Sies et al., 2017). This deterioration reduces the efficiency of energy metabolism mainly by interfering with mitochondrial function (Pan et al., 2008) and causing physical fatigue. Antioxidant capacity comprises both endogenous antioxidants produced within the body and exogenous antioxidants obtained from food, which removes ROS and oxidized biomolecules, facilitating recovery from oxidative damage (Shahidi, 2000; Pisoschi and Pop, 2015). Thus, oxidized biomolecules indicate physiological deterioration, whereas exogenous antioxidant capacity obtained through foraging reflects tolerance to oxidative damage.

In wild birds, oxidative stress is linked to fitness factors such as individual resistance probability (Saino et al., 2011; Noguera et al., 2012; Costantini and Dell’Omo, 2015), reproductive decisions (Costantini et al., 2020), reproductive success (Losdat et al., 2013; Costantini et al., 2015), behavioral traits like migration (Larcombe et al., 2008; Alan and McWilliams, 2013) and foraging behavior (Koyama et al., 2021), as well as external factors such as urbanization, environmental pollution (Espín et al., 2016; Isaksson et al., 2017; Salmón et al., 2018), and pathogens (Costantini and Dell’Omo, 2006; Isaksson et al., 2013; Sebastiano et al., 2017). Therefore, measuring oxidative damage and antioxidant capacity, which may be associated with behavior and fitness, is ideal for quantifying the physiological deterioration and resilience of wild birds. However, few studies have combined detailed measurements of foraging and movement behavior with oxidative stress data in wild animals, and the combination of oxidative stress measurements with precise behavioral tracking enabled by biologging remains particularly rare. As a result, our understanding of the relationship between behavior and oxidative stress remains limited.

Here, we focused on two seabirds, black-tailed gull (*Larus crassirostris*) and streaked shearwater (*Calonectris leucomelas*), which are similar in body size (Chochi et al., 2002; Shirai et al., 2013) and primarily feed on small fish such as anchovies (Narita and Narita, 2004; Kurasawa et al., 2012; Matsumoto et al., 2012; Yoda et al., 2012; Kazama et al., 2018; De Alwis et al., 2025). During the breeding season, we recorded the behavior of these birds for approximately 1 week by using GPS loggers attached to them. Additionally, we measured changes in oxidative stress at the beginning and end of the behavioral recording. Species differences in oxidative stress levels were used as an indicator of chronic physiological stress that is expected to be linked to their habitat, life history, and physiological function. During the breeding season, black-tailed gulls, which often inhabit urban areas (Yoda et al., 2012), typically lay two to three eggs (Tomita and Narita, 2021), whereas the streaked shearwaters found in rural areas lay one egg (Oka et al., 2002; Oka, 2004; Koyama et al., 2024b). During the non-breeding season, black-tailed gulls move around the Japanese Archipelago (Tomita et al., 2015), whereas streaked shearwaters migrate to tropical regions near the equator (Yamamoto et al., 2010). These reproductive or migratory differences may cause differences in oxidative stress levels. The species differences in the changes in oxidative stress levels were also used as a reflection of the accumulation of physiological deterioration or recovery due to the recorded behavior. During the breeding season, black-tailed gulls take round trips from the colony to the foraging site (foraging trip) several times per day and forage in coastal and land areas relatively close to their breeding sites (Yoda et al., 2012; Kazama et al., 2018; Park et al., 2024). In contrast, streaked shearwaters undertake foraging trips lasting from one to 14 days and forage around the colony and in the open ocean (Yoda et al., 2014; Matsumoto et al., 2017; Koyama et al., 2024b). Differences in foraging behavior may lead to species-specific changes in oxidative stress over a weekly timescale. Finally, we examined the relationship between foraging behavior and changes in oxidative stress to evaluate the types of behaviors that affect oxidative stress. For example, increasing the foraging time, during which individuals obtain food, is expected to decrease oxidative stress. Additionally, the influence of foraging and provisioning behaviors on oxidative stress may vary depending on the species. For instance, streaked shearwaters, which adopt energy-saving flight called dynamic soaring (Mir et al., 2018), may not generate ROS compared to black-tailed gulls, which adopt flapping flight, due to differences in heart rate increases associated with these flight styles (Ropert-Coudert et al., 2006; Sakamoto et al., 2013). Thus, it is expected that increasing flight distance would elevate oxidative stress in flapping black-tailed gulls but does not have a significant effect on soaring streaked shearwaters.

## 2 Method

### 2.1 Fieldwork and data collection

Fieldwork was conducted on egg-laying black-tailed gulls in Kabushima Island (40° 32′N, 141° 33′E), during May in 2018, 2019, and 2021 and on chick-rearing streaked shearwaters in Awashima Island (38° 28′N, 139° 14′E), from mid-August to late September from 2018 to 2023. Birds were captured, their blood samples were collected, and GPS loggers were attached to them. Blood samples were collected from the wing veins of the gulls and the lower limb veins of the shearwaters using a needle and syringe prefilled with a small amount of the anticoagulant heparin sodium (5000 units 5 mL^-1^; Mochida Pharmaceutical Co., Ltd., Tokyo, Japan). The volume of collected blood samples was less than 1 mL, corresponding to less than 1% of the body mass of the birds. Blood samples were centrifuged at 2,680 g for 10 min at room temperature, divided into blood cells and plasma, and frozen. Blood sampling did not significantly affect the behavioral parameters (detailed in Supplementary Material, Supplementary Table S1). Body masses of the birds were measured in 5 g units using a spring scale (Pesola LightLine Metric 11,000, ±0.3%; PESOLA AG, Baar, Switzerland) before logger attachment (Supplementary Material, Supplementary Tables S2, S3).

Animal-borne GPS loggers (Axy-Trek with resin 55 × 25 × 11 mm, 25 g; Technosmart, Roma, Italy) were attached on the birds’ back using waterproof tape (Tesa®; Beiersdorf AG, Hamburg, Germany) and cyanoacrylate glue (Henkel Loctite Adhesives Ltd., Hatfield, UK). The Axy-Trek without resin was housed in waterproof heat-shrink tubing (less than 25 g) and attached to the bird’s back using a Teflon ribbon (TH-25; width, 6 mm; Bally Ribbon Mills, Bally, PA, U.S.A.). Another type of GPS logger (PinPoint VHF with solar panels, body size excluding antenna 82 × 25 × 27 mm, 18 g; Lotek Wireless Inc., Newmarket, Ontario, Canada) were attached to the back of the bird using a Teflon ribbon. The PinPoint VHF and Axy-Trek loggers were attached to different individuals. The GPS sampling intervals were set to either 1 point per minute or 1 point every 5 min. The attachment of the same or similarly sized loggers did not significantly affect reproduction (Yoda et al., 2012) or reproduction, behavior, and subsequent survival of the shearwaters (Shiomi et al., 2012; Yoda et al., 2014; Koyama et al., 2024b).

On the day of logger attachment and day after, the birds were recaptured, and additional blood samples were taken and measured body mass after the loggers were retrieved. Individuals with PinPoint VHF loggers were released to record their migration.

The sexes of the birds were determined based on their body size for the gulls (males being larger than females; Chochi et al., 2002), and on their vocalizations for the shearwaters (males had higher-pitched calls than females; Arima et al., 2014). Based on a previous study (Koyama et al., 2021), we included in the analysis individuals that could be recaptured; a second blood sample was collected from these individuals within 17 days after logger attachment and from the date of first blood sampling.

### 2.2 Oxidative stress measurement

Plasma pro-oxidant levels (derivatives of reactive oxygen metabolites; d-ROMs) and plasma antioxidant capacity (biological antioxidant potential; BAP) were measured using a free radical analyzer (FREE Carrio Duo; Diacron International, Grosseto, Italy). The d-ROMs, expressed in Carratelli units (U. CARR = 0.08 mg H_2_O_2_ dL^-1^), reflect plasma concentrations of hydroperoxides (R-OOH) while BAP represents both the endogenous organic compounds and external substances acquired from food. The measurements were conducted according to the experimental protocol described by Koyama et al. (2021). The values of BAP for the gulls in 2018 were measured twice, and the average value was used in the subsequent analysis. Changes in the d-ROMs and BAP levels were calculated by subtracting the levels of the first blood sample (at the time of logger deployment) from those of the second blood sample (at the time of logger retrieval).

### 2.3 Data analysis

Data analyses were conducted using R version 4.2.1 (R Development Core Team, 2020). The duration between the first and second blood sampling did not influence the changes in d-ROMs or BAP (Supplementary Material, Supplementary Table S4). Therefore, the duration of blood sampling was not taken into account in the subsequent analysis. To evaluate the species-based difference in changes in oxidative stress using linear regression models, we used the ‘lm’ function in R, with species as the explanatory variable and changes in d-ROMs or changes in BAP values as the response variables. To examine species differences in d-ROMs and BAP values, we used Tobit models implemented with the ‘vglm’ function in the VGAM package (Yee, 2025). Species was set as the explanatory variable, and d-ROMs and BAP values were set as the response variables. Tobit models are appropriate for modeling response variables with range restrictions. For this analysis, the accurate detection limits of the analyzer, 40 to 1,000 for d-ROMs and 500 to 6,000 for BAP, were set as the detection ranges.

We analyzed the effect of handling time (ranging from 1 to 9 min for black-tailed gulls and 1–32 min for streaked shearwaters in our study) from capture to the completion of blood sampling. Handling times of 10 min or less, which encompassed all samples of black-tailed gulls and 96.8% (179 out of 185) samples of streaked shearwaters, did not significantly affect d-ROMs or BAP values (detailed in the Supplementary Material; Supplementary Tables S5, S6; Supplementary Figure S1). While blood collection is generally recommended to be completed within 3 min, particularly for measuring baseline hormone levels, which are highly sensitive to short-term handling (Kitaysky et al., 1999), previous studies have reported no significant effects on oxidative stress variation in birds even with handling times of 30 min or longer (Costantini et al., 2007; Fowler et al., 2023). Given these findings, the handling times in our study likely had minimal impact on oxidative stress.

The GPS data recorded by the loggers were extracted to match the period between the first and second blood samples. From this dataset, we removed GPS points with ground speeds exceeding 90 km h^-1^ using “ddfilter” function of SDLfilter package (Shimada, 2023) in R. Foraging trip was defined as an extended trip of more than 1 km from the colony and more than 1 h for the gulls (Yoda et al., 2012) and more than 3 km from the colony and more than 6 h (Koyama et al., 2021) for the shearwaters. Foraging trip parameters, such as total flight distance, maximum distance from the colony, and number of takeoffs, were calculated for each foraging trip. To calculate number of takeoffs, flight state was defined as ground speed more than 15 km h^-1^ calculated from GPS points (Shiomi et al., 2012; Yoda et al., 2012). The number of takeoffs was calculated as the number of times the birds switched from the stationary phase (less than 15 km h^-1^) to the flight phase (greater than 15 km h^-1^). We then calculated the average frequency of takeoffs by dividing the number of takeoffs by the trip duration for each foraging trip.

To calculate the foraging duration, we applied hidden Markov models (HMM) to classify bird behavior into three states: resting, foraging, and flight. These states are commonly used to describe the behavior of seabirds that forage exclusively in the ocean, such as shearwaters (Clay et al., 2020; Darby et al., 2022). First, intervals of GPS points were resampled to 10 min intervals using “redisltraj” function in adehabitatLT package (Clement et al., 2024) of R because in previous studies that classified seabird behavior into three states using HMMs, GPS data with 5–30 min intervals were often used (Clay et al., 2020; Darby et al., 2022; Clay et al., 2023; Gillies et al., 2023a; 2023b). Each GPS point was classified into three states based on the step length and turning angle between GPS points using “momentuHMM” function in momentuHMM package (McClintock and Michelot 2022) of R. Step lengths were calculated as the net distance between two consecutive GPS points. Turning angles, representing changes in direction, were calculated as the angles between two consecutive step vectors constructed from successive GPS points. We defined the resting, foraging, and flight states as GPS points with small step lengths and turning angles, medium step lengths and large turning angles, and having large step lengths and small turning angles, respectively. We defined the continuous GPS points classified as a foraging state of more than two as the foraging phase. The foraging duration was calculated by summing the time differences between the first and last GPS points of each foraging phase.

To calculate the foraging parameters for individuals, the total flight distance and number of takeoffs were calculated by summing the values across all foraging trips for each individual. The average maximum distance was calculated by dividing the total maximum distance from the colony, which was calculated by adding the values of each trip, by the number of trips. The average frequency of takeoffs for each individual was calculated by averaging the average frequency of takeoffs for each foraging trips. The percentage of foraging duration was calculated by dividing the foraging duration by the logger-recorded duration. For the gulls that use urban areas to forage, insects, and food derived from human activities on land (Yoda et al., 2012; Mizutani et al., 2021), utilization percentage of the land per individual was calculated. Because GPS points may tend to be missing in urban areas than over the ocean, GPS points were resampled to 1 min intervals using “approx.irts” function in tseries package (Trapletti et al., 2024). We counted GPS points corresponding to flight states, defined as ground speeds greater than 15 km h^-1^ (Shiomi et al., 2012; Yoda et al., 2012), during foraging trips and calculated the utilization percentage of the land divided by all resampled GPS points during foraging trips for individuals.

We established Bayesian regression models using “brms” function in “brms” package (Bürkner et al., 2024) for each year and species to evaluate the relationships between behavioral metrics and oxidative stress. The relationship between changes in oxidative stress and foraging behavior may vary depending on food availability in the surrounding environment (Koyama et al., 2021; Lin et al., 2022), and these relationships were evaluated separately for each year. We used the total flight distance, number of takeoffs, average maximum distance, average frequency of takeoffs, foraging duration, percentage of foraging duration, utilization percentage of the land for the gulls, and sex as explanatory variables. To avoid multicollinearity, variance inflation factors (VIF) were calculated using “vif” function in car package (Fox et al., 2024). The parameters with the highest VIF were removed until the VIF values of all the parameters were less than three (Zuur et al., 2010; Supplementary Table S7). Changes in d-ROMs and BAP were used as response variables. Gaussian distributions were used in this analysis. The relationship between changes in oxidative stress and behavioral parameters was evaluated based on the calculated 95% Bayesian credible interval (CI). A CI greater than zero was considered a positive relationship, and *vice versa*.

## 3 Results

We obtained the oxidative and behavioral data for 41 black-tailed gulls (17, 9, and 15 gulls for 2018, 2019, and 2021, respectively) and 101 streaked shearwaters (11, 20, 18, 17, 23, and 12 shearwaters for 2018, 2019, 2020, 2021, 2022, and 2023, respectively). The average duration between the first and second blood samples was 7.51 days (range, 4–14 days) for the gulls and 5.74 days (range, 1–17 days) for the shearwaters. Because all d-ROMs values of streaked shearwaters in 2022 (range, 3–39 U. CARR) and 2023 (range, 5–33 U. CARR) were lower than the accurate detection limit of the analyzer, 40 U. CARR, we excluded them from the following analysis.

**FIGURE 1:**
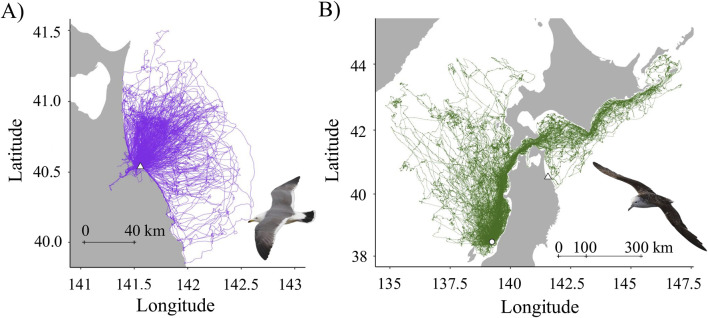
Trajectories of black-tailed gulls **(A)** and streaked shearwaters **(B)**. Purple lines represent the flight trajectories of black-tailed gulls, whereas green lines represent those of streaked shearwaters. Triangle and circle marks indicate the breeding site of black-tailed gulls, Kabushima Island, and that of streaked shearwaters, Awashima Island, respectively. The copyrights of the seabird photos in Figures 1A, B belong to Yuichi Mizutani and Yusuke Goto, respectively, who are authors of this paper.

We recorded 400 and 337 foraging trips for the gulls (Figure 1A) and the shearwaters (Figure 1B), respectively. At the individual scale, for the gulls, total trip duration was 71.21 ± 31.99 (h), total flight distance was 923.48 ± 454.31 (km), average maximum distance from the colony was 33.8 ± 13.54 (km), average frequency of takeoffs was 0.81 ± 0.32 (times h^-1^), foraging duration was 43.84 ± 22.35 (h), percentage of foraging duration was 23.84 ± 8.78 (%), and utilization percentage of the land was 11.02 ± 13.62 (%). For the shearwaters, total trip duration was 121.06 ± 98.69 (h), total flight distance was 1,651.49 ± 1,496.27 (km), average maximum distance from the colony was 221.63 ± 201.39 (km), average frequency of takeoffs was 2.08 ± 0.69 (times h^-1^), foraging duration was 45.36 ± 36.74 (h), and percentage of foraging duration for individuals was 37.87 ± 11.87 (%).

**FIGURE 2:**
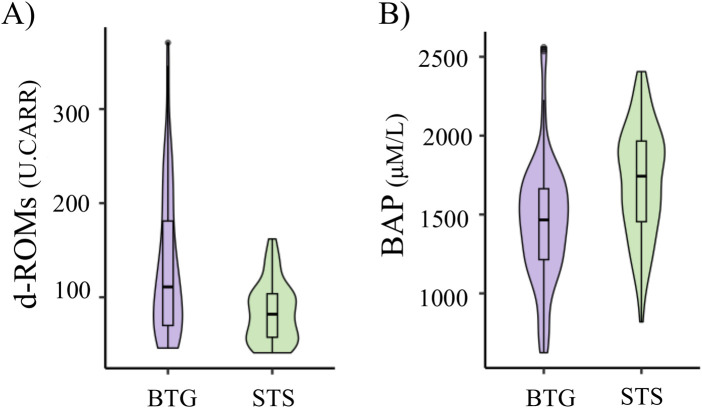
Species differences in d-ROMs **(A)** and BAP **(B)**. The values of black-tailed gulls (BTG) are represented by the purple violin plot, whereas those of streaked shearwaters (STS) are represented by the green violin plot. The d-ROMs values are expressed in Carratelli units (U. CARR = 0.08 mg H_2_O_2_ dL^-1^).

For black-tailed gulls, the average d-ROMs, BAP, changes in d-ROMs, changes in BAP were 131.52 ± 76.52 (U. CARR) (average ±standard deviation), 1,437.51 ± 416.73 (μM/L), −10.67 ± 71.27, and 44.87 ± 498.76, respectively (Supplementary Table S8A). For streaked shearwaters, the average d-ROMs, BAP, changes in d-ROMs, changes in BAP were 59.61 ± 36.59 (U. CARR), 1706 ± 352.85 (μM/L), −6.37 ± 39.92, −97.88 ± 365.04, respectively (Supplementary Table S8B). d-ROMs was significantly larger in gulls than in shearwaters (estimate = −49.01; P < 0.01, Figure 2A). BAP was significantly lower in gulls than in shearwaters (estimate = −252, P < 0.01; Figure 2B). There were no significant differences between the changes in d-ROMs (estimate = 4.30, P = 0.69) and BAP (estimate = −142.74, P = 0.093) for gulls and shearwaters.

Using the brms, we evaluated the relationship between behavioral parameters and changes in oxidative stress each year. For the gulls, changes in d-ROMs were negatively related to the percentage of foraging duration (−14.48 [95% CI –27.08, −2.00]) and the utilization percentage of the land (−5.19 [95% CI –10.02, −0.25]) in 2018, and positively related to the total flight distance (0.06 [95% CI 0.01, 0.12]) in 2021 (Table 1; Supplementary Table S7A). For the shearwaters, changes in BAP were positively related to the foraging duration (5.83 [95% CI 1.69, 9.88]) and negatively related to the average maximum distance (−0.69 [95% CI –1.26, −0.13]) in 2022 and negatively related to the average frequency of takeoffs (−458.47 [95% CI –747.12, −152.72]) in 2023. Sex-based differences in the changes in BAP were observed in 2023; the changes in males were larger than those in females (395.26 [95% CI 146.58, 630.01]) for the shearwaters (Table 1; Supplementary Table S7B). There was no significant relationship between the changes in oxidative stress and behavioral parameters for the gulls in 2019, and for the shearwaters from 2018 to 2021.

**TABLE 1:** Relationship between behavioral parameters and the changes in d-ROMs of black-tailed gulls (A) and changes in BAP of streaked shearwaters (B). Only the years with significant relationships are presented. Behavioral parameters removed based on VIF are indicated by blank spaces. N.S. indicates no significant relationship. Positive (+) and negative (−) relationships are shown where applicable.

(A)Year	2018	2021
total flight distance	N.S.	+
average maximum distance	N.S.	N.S.
foraging duration		
percentage of foraging duration	–	N.S.
average frequency of takeoffs	N.S.	
utilization percentage of the land	–	N.S.
sex (male)	N.S.	N.S.

(B)Year	2022	2023
total flight distance		
average maximum distance	–	
foraging duration	+	N.S.
percentage of foraging duration		N.S.
average frequency of takeoffs	N.S.	–
sex (male)	N.S.	+

## 4 Discussion

Black-tailed gulls during the egg-laying period exhibit higher oxidative damage and lower antioxidant capacity than streaked shearwaters during the chick-rearing period in terms of chronic oxidative stress. The species differences might be explained by differences in their habitat, species-specific physiological functions, and reproductive stages. As environmental pollution (Espín et al., 2016; Isaksson et al., 2017; Salmón et al., 2018) and pathogens (Costantini and Dell’Omo, 2006; Isaksson et al., 2013; Sebastiano et al., 2017) can increase oxidative stress, living in urban areas might increase oxidative stress in black-tailed gulls. Black-tailed gulls, which inhabit a variety of environments, might possess physiological mechanisms that enhance their tolerance to oxidative damage as well as antioxidant capacity, such as autophagy (promoting the degradation of damaged biomolecules) (Yun et al., 2020) and DNA repair pathways (Barzilai and Yamamoto, 2004), preventing oxidative damage from becoming fatal. In contrast, streaked shearwaters exhibit reproductive traits, with their chicks growing slowly and storing fat reserves to withstand starvation (Oka et al., 2002). Previous studies have shown that birds with higher fat content have been found to possess higher antioxidant capacities (Costantini et al., 2007 but see Eikenaar et al., 2020), and it is possible that adult streaked shearwaters, displaying similar traits as their chicks, may also store fat and similarly accumulate antioxidant compounds in their bodies, enhancing their tolerance to oxidative damage. In 2022 and 2023, the oxidative damage to the shearwaters was so low that it fell below the detection limit of the measurement equipment. As shown in other migratory birds, streaked shearwaters, which migrate approximately 3,000 km from Japan to the equator (Takahashi et al., 2008), might accumulate antioxidant capacity in preparation for extended migrations (Skrip and Mcwilliams, 2016; Gutiérrez et al., 2019), or may have evolved mechanisms to upregulate endogenous antioxidant capacity in response to elevated oxidative damage (Costantini, 2008; Jenni-Eiermann et al., 2014; Cooper-Mullin and McWilliams, 2016). Notably, our results did not confirm the existence of these physiological functions in either species. We encourage further studies to clarify species-specific physiological mechanisms and their adaptations to habitat and life histories. Furthermore, the reproductive stage is also a factor that may influence oxidative stress in birds and mammals, including seabirds (Costantini et al., 2014; Montoya et al., 2016; Speakman and Garratt, 2014). As demonstrated in previous studies, incubation might increase oxidative stress in gulls compared to chick-rearing, although the underlying mechanism remains unclear (Colominas-Ciuró et al., 2017; 2022; Kulaszewicz et al., 2018). We recommend that future studies investigate whether reproductive factors, including not only reproductive stage but also egg number, egg size, chick condition, and chick age, are associated with parental oxidative stress, and explore the mechanisms driving these relationships.

Over a weekly timescale, there were no significant species differences in changes in oxidative damage and antioxidant capacity, suggesting that behavioral differences, such as daily short-range coastal foraging *versus* extended oceanic foraging trips lasting up to 2 weeks, might not necessarily lead to species-specific changes in oxidative stress. Our findings indicate that seabirds might possess physiological adaptations to prevent oxidative stress buildup over a weekly timescale, even during the breeding season, which is one of their most energetically demanding periods. These adaptations may include physiological mechanisms such as the upregulation of nuclear factors that modulate antioxidant defenses in response to oxidative damage, similar to those observed in laboratory and domestic animals, as well as humans (Ungvari et al., 2008; Ge et al., 2019; Surai et al., 2019; Castiglione et al., 2020).

There was no consistent trend in the relationship between oxidative stress and behavioral metrics across all years or species, suggesting that the effects of behavior on oxidative stress may vary with environmental differences each year, regardless of species. The energy requirements for flight may vary with annual wind conditions (Furness and Bryant, 1996; Thorne et al., 2023), and the effect of migration on oxidative stress may fluctuate. Additionally, as shown in black-tailed gulls in 2018 and streaked shearwaters in 2022, a longer foraging duration might decrease oxidative damage or increase antioxidant capacity resulting in decreased oxidative stress in years when food availability is high. However, a longer foraging duration might not result in obtaining food, which decreases oxidative stress, depending on the food species that change from year to year (De Alwis et al., 2025), food availability (Garthe et al., 2011; Horswill et al., 2017), and the presence of competitors (Koyama et al., 2024a). Our results indicate no significant species differences in the changes in oxidative stress, species-related factors such as flight style, reproductive traits, and life history might not influence oxidative stress as strongly as environmental factors.

Comprehensive investigations of behavioral and physiological conditions offer significant insights into how wild animals adapt to their habitats through species-specific functions and provide a foundation for assessing species’ vulnerabilities and responses to threats such as urbanization and climate change (Cooke et al., 2014; Andrews and Enstipp, 2016). To date, comprehensive investigations have mainly been conducted on large marine animals, such as seals and turtles, by combining multiple loggers that record their behavioral activity and physiological conditions, such as heart rate (Ropert-Coudert et al., 2006; Clark et al., 2010; Viblanc et al., 2011; McDonald et al., 2018), body temperature (Southwood et al., 2005; Papastamatiou et al., 2015), and brain activity (Kendall-Bar et al., 2023). Unlike logger-based physiological recordings, measurement of oxidative damage allows for a more direct assessment of physiological stress in animals by quantifying the damage to biomolecules. Additionally, antioxidant capacity reflects the quality of an animal’s food intake and nutritional status, providing valuable insights into the physiological effects of external factors such as food availability. Thus, we propose combining behavioral biologging with oxidative stress measurement, which is useful for quantifying oxidative damage as physiological deterioration caused by exercise and antioxidant capacity as tolerance to oxidative damage derived from the prey, despite its limitation in providing only instantaneous physiological data. Integrating environmental, behavioral, and oxidative stress data would help identify the environmental factors that cause physiological deterioration or recovery, especially for small- and medium-sized animals, for which attaching multiple loggers is challenging owing to weight limitations (Barron et al., 2010; Portugal and White, 2018). Oxidative stress measurements have been measured in marine animals of various sizes, including seals (Vázquez-Medina et al., 2012), sea turtles (Labrada-Martagón et al., 2011; Perrault and Stacy, 2018), seabirds (Merkling et al., 2017; Kulaszewicz et al., 2018), sharks (Barrera-García et al., 2012; Vélez-Alavez et al., 2013), and fish (Janssens et al., 2000; Bacanskas et al., 2004; Cappello et al., 2016), demonstrating its potential for broad applications across marine species. We encourage future integrated studies on the marine environment, behavior, and oxidative stress of different species and animals to clarify what kind of foraging and provisioning behavior contributes to either the deterioration or recovery of their physiological conditions depending on the surrounding environment.

## Data Availability

The original contributions presented in the study are included in the article/Supplementary Material, further inquiries can be directed to the corresponding author.
