# Supplementary material for: Species-specific physiological status in seabirds: insights from integrating oxidative stress measurements and biologging
Source: Front Physiol. 2025 Mar 19;16:1509511. doi: 10.3389/fphys.2025.1509511 (PMC11962040; doi:10.3389/fphys.2025.1509511)
Supplement: Supplementary file 1 [file Supplementaryfile1.docx]

**Supplementary materials**

To evaluate the effect of blood drawing on the basic behavioral parameters, we established Bayesian regression models (BRM) using “brms” function in brms package for each species. Blood sampling as a categorical explanatory variable and individual ID and year as random effects. Trip duration, total flight distance, and maximum distance from the colony for each foraging trip were used as response variables. All behavioral parameters used as response variables were log transformed, and a normal distribution was used as the probability distribution. Recorded trip numbers were 400, 952, 337, and 1410 for gulls with blood sampling, gulls without blood sampling, shearwaters with blood sampling, and the shearwaters without blood sampling, respectively. The relationship was evaluated based on the calculated 95% Bayesian credible interval (CI). A CI greater than zero was considered a positive relationship, and vice versa. As a result, there was no significant effect of blood sampling on basic behavioral parameters in both species (Table S1).

To gain a basic understanding of the relationship between oxidative stress and body mass, we established BRM for each species. We used the d-ROMs and BAP as response variables. and body mass and sex as explanatory variables. Year was included as a random effect. Gaussian distributions were applied in this analysis. As a result, body mass showed no significant relationships with d-ROMs and BAP levels in either species (Table S2). To evaluate the relationship between the changes in oxidative stress and changes in body mass, we used the changes in d-ROMs and BAP as response variables. Changes in body mass and sex were used as explanatory variables, with year as a random effect. Gaussian distributions were applied in this analysis. As a result, changes in body mass showed no significant relationships with changes in d-ROMs and BAP in either species (Table S3).

We established BRM for each species to evaluate whether the duration between the first and second blood samplings influenced changes in oxidative stress. Changes in d-ROMs and BAP were used as response variables. The duration of blood drawings and sex were used as explanatory variables. Year was used as a random effect. Gaussian distributions were applied in this analysis. As a result, there was no significant relationship between the duration and changes in oxidative stress (Table S4).

To evaluate the effect of handling time (ranging from 1 to 32 minutes in our study) from capture to the completion of blood sampling on oxidative stress, we used Tobit models with “vglm” function in the VGAM package for each species. Handling time and year set as explanatory variables, and d-ROMs and BAP values were set as the response variables. The accurate detection limits of the analyzer, 40 to 1000 for d-ROMs and 500 to 6000 for BAP, were set as the detection ranges in the Tobit models. Handling time were recorded for 40 blood samples from black-tailed gulls (16 and 24 samples for 2019 and 2021, respectively) and 185 blood samples from streaked shearwaters (19, 35, 34, 34, 42, and 21 samples for 2018, 2019, 2020, 2021, 2022, and 2023, respectively). As a result, there were no significant relationships between handling time and d-ROMs values in shearwaters, d-ROMs values in gulls, and BAP values in gulls (Table S5, Figure S1). There was significant relationship between the handling time and BAP values of the shearwaters (Table S5B), while in the dataset with handling times of 10 minutes or less—accounting for 96.8% (179 out of 185) of streaked shearwater samples—handling time did not significantly affect BAP values in shearwaters (Table S6).

Table S1: There were no effects of blood drawing on behavioral parameters, trip duration, total flight distance, and maximum distance from the colony for each foraging trip of black tailed gulls (A) and that of streaked shearwaters (B).

(A)

| Response variables | Explanatory variables | Estimate | Estimate Error | L-95% CI | U-95% CI | Rhat |
| --- | --- | --- | --- | --- | --- | --- |
| trip duration | intercept | 7.49 | 4.02 | -0.28 | 15.55 | 1 |
|  | blood drawing (without) | 1.67 | 3.17 | -4.61 | 7.8 | 1 |
| total flight distance | intercept | 96.06 | 28.38 | 38.17 | 158.86 | 1.01 |
|  | blood drawing (without) | 1.41 | 15.78 | -29.33 | 31.75 | 1 |
| maximum distance from the colony | intercept | 32.96 | 6.51 | 18.47 | 46.25 | 1 |
|  | blood drawing (without) | -2.01 | 3.14 | -8.36 | 3.98 | 1 |

(B)

| Response variables | Explanatory variables | Estimate | Estimate Error | L-95% CI | U-95% CI | Rhat |
| --- | --- | --- | --- | --- | --- | --- |
| trip duration | intercept | 54.37 | 7.44 | 39.26 | 68.53 | 1 |
|  | blood drawing (without) | -0.13 | 3.73 | -7.31 | 7.18 | 1 |
| total flight distance | intercept | 729.8 | 114.81 | 498.12 | 949.05 | 1 |
|  | blood drawing (without) | -22.26 | 50.52 | -124.66 | 75.89 | 1 |
| maximum distance from the colony | intercept | 208.31 | 30.95 | 146.53 | 271.41 | 1 |
|  | blood drawing (without) | -7.8 | 15.2 | -37.48 | 21.78 | 1 |

Table S2: Relationship between the oxidative stress levels and body mass of black tailed gulls (A) and that of streaked shearwaters (B).

(A)

| Response variables | Explanatory variables | Estimate | Estimate Error | L-95% CI | U-95% CI | Rhat |
| --- | --- | --- | --- | --- | --- | --- |
| d-ROMs | intercept | 108.13 | 90.7 | -65.51 | 289.59 | 1 |
|  | body mass | 0.02 | 0.14 | -0.25 | 0.29 | 1 |
|  | sex | 1.23 | 20.12 | -37.4 | 40.17 | 1 |
| BAP | intercept | 1510.56 | 599.86 | 319.64 | 2650.76 | 1 |
|  | body mass | 0.09 | 1.13 | -2.05 | 2.29 | 1 |
|  | sex | -183.33 | 156.84 | -488.15 | 120.55 | 1 |

(B)

| Response variables | Explanatory variables | Estimate | Estimate Error | L-95% CI | U-95% CI | Rhat |
| --- | --- | --- | --- | --- | --- | --- |
| d-ROMs | intercept | 116.09 | 34.94 | 46.48 | 185.25 | 1 |
|  | body mass | -0.1 | 0.06 | -0.21 | 0.02 | 1 |
|  | sex | 7.37 | 7.83 | -8.31 | 22.64 | 1 |
| BAP | intercept | 1593.79 | 250.81 | 1081.92 | 2084.65 | 1 |
|  | body mass | 0.22 | 0.45 | -0.69 | 1.1 | 1 |
|  | sex | -6.18 | 56.71 | -119.63 | 101.65 | 1 |

Table S3: Relationship between the changes in oxidative stress and the changes in body mass of black tailed gulls (A) and that of streaked shearwaters (B).

(A)

| Response variables | Explanatory variables | Estimate | Estimate Error | L-95% CI | U-95% CI | Rhat |
| --- | --- | --- | --- | --- | --- | --- |
| changes in d-ROMs | intercept | -33.87 | 53.2 | -133.97 | 72.5 | 1 |
|  | changes in body mass | 2.63 | 5.7 | -8.46 | 13.7 | 1 |
|  | sex | 8.63 | 25.16 | -40.41 | 58.25 | 1 |
| changes in BAP | intercept | -99.62 | 51.93 | -199.65 | 4.87 | 1 |
|  | changes in body mass | 1.08 | 0.77 | -0.41 | 2.61 | 1 |
|  | sex | 48.33 | 66.25 | -80.13 | 176.55 | 1 |

(B)

| Response variables | Explanatory variables | Estimate | Estimate Error | L-95% CI | U-95% CI | Rhat |
| --- | --- | --- | --- | --- | --- | --- |
| changes in d-ROMs | intercept | -10.3 | 11.85 | -33.14 | 14.57 | 1 |
|  | changes in body mass | -0.19 | 0.12 | -0.43 | 0.05 | 1 |
|  | sex | -0.5 | 10.43 | -20.68 | 19.82 | 1 |
| changes in BAP | intercept | -99.62 | 51.93 | -199.65 | 4.87 | 1 |
|  | changes in body mass | 1.08 | 0.77 | -0.41 | 2.61 | 1 |
|  | sex | 48.33 | 66.25 | -80.13 | 176.55 | 1 |

Table S4: Relationship between the changes in oxidative stress and duration between the first and second blood drawings of black tailed gulls (A) and that of streaked shearwaters (B).

(A)

| Response variables | Explanatory variables | Estimate | Estimate Error | L-95% CI | U-95% CI | Rhat |
| --- | --- | --- | --- | --- | --- | --- |
| changes in d-ROMs | intercept | -33.87 | 53.2 | -133.97 | 72.5 | 1 |
|  | blood duration | 2.63 | 5.7 | -8.46 | 13.7 | 1 |
|  | sex | 8.63 | 25.16 | -40.41 | 58.25 | 1 |
| changes in BAP | intercept | 76.59 | 364.8 | -644.66 | 773.61 | 1 |
|  | blood duration | -31.45 | 37.76 | -104.25 | 43.74 | 1 |
|  | sex | 281.45 | 169.89 | -48.89 | 612.47 | 1 |

(B)

| Response variables | Explanatory variables | Estimate | Estimate Error | L-95% CI | U-95% CI | Rhat |
| --- | --- | --- | --- | --- | --- | --- |
| changes in d-ROMs | intercept | -12.45 | 13.87 | -39.65 | 15.57 | 1 |
|  | blood duration | 0.79 | 1.22 | -1.6 | 3.2 | 1 |
|  | sex | 1.21 | 9.93 | -18.46 | 21.01 | 1 |
| changes in BAP | intercept | -6.29 | 8.02 | -21.95 | 10.13 | 1 |
|  | blood duration | 0.32 | 0.79 | -1.24 | 1.88 | 1 |
|  | sex | 1.52 | 6.55 | -10.92 | 14.36 | 1 |

Table S5: Relationship between handling time and oxidative stress of black tailed gulls (A) and that of streaked shearwaters (B).

(A)

| Response variables | Explanatory variables | Estimate | Std. Error | z value | p value |
| --- | --- | --- | --- | --- | --- |
| d-ROMs | intercept1 | 1511 | 189.74 | 7.97 | <0.00 |
|  | intercept2 | 5.73 | 0.11 | 51.21 | <0.00 |
|  | Handling Time | -8.65 | 32.63 | -0.27 | 0.79 |
|  | Year (2021) | -71.31 | 135.43 | -0.53 | 0.6 |
| BAP | intercept1 | 59.54 | 18.81 | 3.17 | 0.0016 |
|  | intercept2 | 3.4 | 0.12 | 28.26 | <0.00 |
|  | Handling Time | 2.95 | 3.23 | 0.91 | 0.36 |
|  | Year (2021) | 22.13 | 13.45 | 1.65 | 0.01 |

(B)

| Response variables | Explanatory variables | Estimate | Std. Error | z value | p value |
| --- | --- | --- | --- | --- | --- |
| d-ROMs | intercept1 | 103.29 | 7.69 | 13.43 | <0.00 |
|  | intercept2 | 3.27 | 0.091 | 36.07 | <0.00 |
|  | Handling Time | 0.7 | 0.77 | 0.91 | 0.36 |
|  | Year (2019) | -29.8 | 9.91 | -3.01 | 0.0026 |
|  | Year (2020) | -36.83 | 9.12 | -4.04 | <0.00 |
|  | Year (2021) | -33.07 | 8.41 | -3.93 | <0.00 |
| BAP | intercept1 | 1908 | 70.23 | 27.17 | <0.00 |
|  | intercept2 | 5.58 | 0.052 | 107.24 | <0.00 |
|  | Handling Time | -19.46 | 75.81 | -0.77 | 0.00084 |
|  | Year (2019) | -58.4 | 75.81 | -0.77 | 0.44 |
|  | Year (2020) | -439.37 | 76.43 | -5.75 | <0.00 |
|  | Year (2021) | -309.74 | 75.92 | -4.08 | <0.00 |
|  | Year (2022) | 161.13 | 73.76 | 2.19 | 0.029 |
|  | Year (2023) | 76.32 | 85.01 | 0.9 | 0.37 |

Table S6: Relationship between handling time 10 minutes or less and BAP of streaked shearwaters.

| Response variables | Explanatory variables | Estimate | Std. Error | z value | p value |
| --- | --- | --- | --- | --- | --- |
| BAP | intercept1 | 1884 | 82.2 | 22.92 | <0.00 |
|  | intercept2 | 5.58 | 0.053 | 105.55 | <0.00 |
|  | Handling Time | -12.25 | 9.73 | -1.26 | 0.21 |
|  | Year (2019) | -81.63 | 78.28 | -1.04 | 0.3 |
|  | Year (2020) | -445.58 | 78.26 | -5.69 | <0.00 |
|  | Year (2021) | -307.78 | 78.05 | -3.94 | <0.00 |
|  | Year (2022) | 159.88 | 76.15 | 2.1 | 0.036 |
|  | Year (2023) | 75.5 | 87.41 | 0.86 | 0.39 |

Table S7: VIF and results of brms analysis of black tailed gulls (A) and that of streaked shearwaters (B).

(A)

| Year | Response variables | Explanatory variables | VIF | Estimate | Estimate Error | L-95% CI | U-95% CI | Rhat |
| --- | --- | --- | --- | --- | --- | --- | --- | --- |
| 2018 | changes in d-ROMs | intercept |  | 85.16 | 158.29 | –221.99 | 405.07 | 1 |
|  |  | total flight distance | 1.64 | 0.01 | 0.09 | –0.17 | 0.19 | 1 |
|  |  | average maximum distance | 1.67 | –0.58 | 4.31 | –9.26 | 7.7 | 1 |
|  |  | percentage of foraging duration | 2.47 | –14.48 | 6.21 | –27.08 | –2.00 | 1 |
|  |  | average number of takeoffs | 2.24 | 348.29 | 215.16 | –87.06 | 780.7 | 1 |
|  |  | utilization percentage of the land | 1.73 | –5.19 | 2.46 | –10.02 | –0.25 | 1 |
|  |  | sex male |  | 9.8 | 62.69 | –111.82 | 133.24 | 1 |
|  | changes in BAP | intercept |  | –1173.83 | 1215.41 | –3549.06 | 1271.56 | 1 |
|  |  | total flight distance | 1.64 | 0.15 | 0.67 | –1.19 | 1.52 | 1 |
|  |  | average maximum distance | 1.67 | 26.87 | 33.32 | –42.44 | 92.32 | 1 |
|  |  | percentage of foraging duration | 2.47 | –7.88 | 44.93 | –93.32 | 80.37 | 1 |
|  |  | average number of takeoffs | 2.24 | 449.08 | 1579.51 | –2761.92 | 3513.39 | 1 |
|  |  | utilization percentage of the land | 1.73 | 11.55 | 18.28 | –23.30 | 47.96 | 1 |
|  |  | sex male |  | 258.12 | 447.26 | –619.73 | 1139.94 | 1 |
| 2019 | changes in d-ROMs | intercept |  | –168.57 | 372.59 | –947.63 | 520.53 | 1.02 |
|  |  | total flight distance | 1.39 | –0.05 | 0.07 | –0.20 | 0.08 | 1.01 |
|  |  | average maximum distance | 1.08 | 3.54 | 5.37 | –6.40 | 14.77 | 1.02 |
|  |  | percentage of foraging duration | 1.56 | –0.96 | 4.69 | –10.24 | 7.85 | 1.01 |
|  |  | utilization percentage of the land | 1.3 | –0.47 | 9.1 | –17.41 | 19.63 | 1.01 |
|  |  | sex male |  | 169.09 | 180.22 | –166.59 | 553.24 | 1.02 |
|  | changes in BAP | intercept |  | 1184.62 | 6650.57 | –9758.34 | 12489.05 | 1.02 |
|  |  | total flight distance | 1.39 | –0.37 | 1.28 | –2.24 | 1.62 | 1.01 |
|  |  | average maximum distance | 1.08 | –14.96 | 95.04 | –176.24 | 134.66 | 1.02 |
|  |  | percentage of foraging duration | 1.56 | 15.17 | 94.94 | –122.08 | 150.57 | 1.01 |
|  |  | utilization percentage of the land | 1.3 | –28.05 | 161.39 | –298.84 | 253.1 | 1.01 |
|  |  | sex male |  | –694.91 | 3343.9 | –6110.79 | 4681.86 | 1.02 |
| 2021 | changes in d-ROMs | intercept |  | –54.98 | 41.64 | –139.16 | 27.43 | 1 |
|  |  | total flight distance | 1.73 | 0.06 | 0.03 | 0.01 | 0.12 | 1 |
|  |  | average maximum distance | 1.55 | –0.80 | 0.82 | –2.37 | 0.87 | 1 |
|  |  | percentage of foraging duration | 1.06 | –0.63 | 0.98 | –2.55 | 1.34 | 1 |
|  |  | utilization percentage of the land | 1.7 | –8.40 | 30.63 | –69.01 | 54.18 | 1 |
|  |  | sex male |  | 36.01 | 26.06 | –16.79 | 88.56 | 1 |
|  | changes in BAP | intercept |  | 108.36 | 664.18 | –1223.72 | 1443.7 | 1 |
|  |  | total flight distance | 1.73 | –0.09 | 0.5 | –1.08 | 0.9 | 1 |
|  |  | average maximum distance | 1.55 | 2.96 | 13.65 | –23.7 | 29.23 | 1 |
|  |  | percentage of foraging duration | 1.06 | 7.05 | 15.06 | –37.74 | 23.77 | 1 |
|  |  | utilization percentage of the land | 1.7 | –189.32 | 508.36 | –1204.88 | 847.38 | 1 |
|  |  | sex male |  | 351.76 | 419.41 | –504.45 | 1158.39 | 1 |

(B)

| Year | Response variables | Explanatory variables | VIF | Estimate | Estimate Error | L-95% CI | U-95% CI | Rhat |
| --- | --- | --- | --- | --- | --- | --- | --- | --- |
| 2018 | changes in d-ROMs | intercept |  | 17.13 | 81.94 | –141.20 | 182.38 | 1 |
|  |  | total flight distance | 1.36 | –0.01 | 0.02 | –0.05 | 0.04 | 1 |
|  |  | average maximum distance | 1.24 | –0.08 | 0.21 | –0.46 | 0.33 | 1 |
|  |  | percentage of foraging duration | 1.12 | 0.79 | 0.79 | 2.66 | 6.07 | 1 |
|  |  | sexM |  | –0.18 | 35.79 | –74.00 | 67.41 | 1 |
|  | changes in BAP | intercept |  | –620.32 | 1317.86 | –3372.28 | 1981.39 | 1 |
|  |  | total flight distance | 1.36 | 0.04 | 0.37 | –0.63 | 0.77 | 1 |
|  |  | average maximum distance | 1.24 | 1.52 | 3.23 | –4.96 | 7.72 | 1 |
|  |  | percentage of foraging duration | 1.12 | 1.94 | 41.61 | –81.92 | 79.73 | 1 |
|  |  | sexM |  | 127.21 | 550.31 | –994.39 | 1208.73 | 1 |
| 2019 | changes in d-ROMs | intercept |  | –66.10 | 83.74 | –235.73 | 92.23 | 1 |
|  |  | average maximum distance | 1.39 | 0.09 | 0.07 | –0.04 | 0.23 | 1 |
|  |  | percentage of foraging duration | 1.78 | 1.19 | 2.2 | –3.02 | 5.8 | 1 |
|  |  | foraging duration | 1.1 | –0 | 0.32 | –0.63 | 0.66 | 1 |
|  |  | average number of takeoffs | 1.43 | –3.34 | 28.15 | –58.19 | 53.52 | 1 |
|  |  | sexM |  | 11.37 | 24.59 | –37.81 | 59.98 | 1 |
|  | changes in BAP | intercept |  | 732.4 | 574.61 | –433.4 | 1842.75 | 1 |
|  |  | average maximum distance | 1.39 | –0.13 | 0.46 | –1.07 | 0.76 | 1 |
|  |  | percentage of foraging duration | 1.78 | –19.21 | 15.41 | –50.26 | 10.97 | 1 |
|  |  | foraging duration | 1.1 | –2.82 | 2.17 | –7.09 | 1.52 | 1 |
|  |  | average number of takeoffs | 1.43 | 27.9 | 194.01 | –350.22 | 409.09 | 1 |
|  |  | sexM |  | 59.58 | 169.13 | –271.55 | 394.81 | 1 |
| 2020 | changes in d-ROMs | intercept |  | –14.40 | 63.72 | –140.32 | 115.67 | 1 |
|  |  | total flight distance | 1.89 | 0 | 0.01 | –0.02 | 0.03 | 1 |
|  |  | average maximum distance | 2.42 | 0.07 | 0.11 | –0.16 | 0.3 | 1 |
|  |  | percentage of foraging duration | 1.34 | 0.03 | 1.23 | –2.41 | 2.46 | 1 |
|  |  | average number of takeoffs | 2.27 | –9.92 | 27.85 | –65.54 | 45.2 | 1 |
|  |  | sexM |  | –2.44 | 26.78 | –56.54 | 51.34 | 1 |
|  | changes in BAP | intercept |  | –249.24 | 466.93 | –1181.44 | 678.16 | 1 |
|  |  | total flight distance | 1.89 | –0.12 | 0.08 | –0.29 | 0.05 | 1 |
|  |  | average maximum distance | 2.42 | 0.39 | 0.87 | –1.26 | 2.13 | 1 |
|  |  | percentage of foraging duration | 1.34 | –3.76 | 9.24 | –22.11 | 14.69 | 1 |
|  |  | average number of takeoffs | 2.27 | 239.98 | 213.53 | –175.6 | 668.77 | 1 |
|  |  | sexM |  | –117.27 | 196.15 | –505.13 | 260.83 | 1 |
| 2021 | changes in d-ROMs | intercept |  | –24.94 | 86.7 | –200.56 | 146.23 | 1 |
|  |  | total flight distance | 2.79 | –0.01 | 0.01 | –0.03 | 0.01 | 1 |
|  |  | average maximum distance | 2.7 | 0.06 | 0.11 | –0.16 | 0.27 | 1 |
|  |  | percentage of foraging duration | 1.26 | –1.77 | 1.87 | –5.59 | –5.59 | 1 |
|  |  | average number of takeoffs | 1.38 | 43.42 | 37.2 | –30.35 | 117.95 | 1 |
|  |  | sexM |  | –11.00 | 29.44 | –68.34 | 46.89 | 1 |
|  | changes in BAP | intercept |  | –1292.83 | 862.24 | –2938.32 | 410.16 | 1 |
|  |  | total flight distance | 2.79 | 0.01 | 0.11 | –0.21 | 0.23 | 1 |
|  |  | average maximum distance | 2.7 | –0.04 | 1.06 | –2.10 | 2.06 | 1 |
|  |  | percentage of foraging duration | 1.26 | 19.42 | 17.95 | –15.50 | 56.45 | 1 |
|  |  | average number of takeoffs | 1.38 | 125.69 | 370.04 | –605.69 | 855.6 | 1 |
|  |  | sexM |  | 560.03 | 288.6 | –2.02 | 1124.68 | 1 |
| 2022 | changes in BAP | intercept |  | –188.88 | 120.29 | –424.78 | 47.98 | 1 |
|  |  | average maximum distance | 2.17 | –0.69 | 0.28 | –1.26 | –0.13 | 1 |
|  |  | foraging duration | 2.2 | 5.83 | 2.03 | 1.69 | 9.88 | 1.01 |
|  |  | average number of takeoffs | 1.04 | –4.00 | 39.58 | –83.99 | 74.08 | 1 |
|  |  | sexM |  | 144.35 | 97.49 | –50.00 | 344.34 | 1 |
|  | changes in BAP | intercept |  | 135.99 | 232.01 | –321.08 | 561.89 | 1 |
|  |  | percentage of foraging duration | 2.35 | 7.43 | 5.39 | –3.19 | 18.07 | 1 |
|  |  | foraging duration | 1.97 | 7.94 | 4.32 | –1.02 | 16.38 | 1 |
|  |  | average number of takeoffs | 2.44 | –458.47 | 150.21 | –747.12 | –152.71 | 1 |
|  |  | sexM |  | 395.26 | 128.2 | 146.58 | 630.01 | 1 |

Table S8: Average, minimum, and maximum values of changes in d-ROMs and BAP in each year for the gulls (A) and for the shearwaters (B).

(A)

| Year | Parameter | Average | Minimum | Maximum |
| --- | --- | --- | --- | --- |
| 2018 | dROMs | –16.26 | –165 | 181.5 |
|  | BAP | 171.97 | –883.5 | 1044.5 |
| 2019 | dROMs | 22 | –58 | 129 |
|  | BAP | –129.56 | –700 | 237 |
| 2021 | dROMs | –23.93 | –87 | 22 |
|  | BAP | 5.47 | –700 | 496 |

(B)

| Year | Parameter | Average | Minimum | Maximum |
| --- | --- | --- | --- | --- |
| 2018 | dROMs | 7.82 | –33 | 63 |
|  | BAP | –106.73 | –903 | 506 |
| 2019 | dROMs | 8.80 | –110 | 89 |
|  | BAP | –106.7 | –631 | 424 |
| 2020 | dROMs | –24.18 | –83 | 22 |
|  | BAP | 0.18 | –492 | 547 |
| 2021 | dROMs | –15.59 | –94 | 69 |
|  | BAP | –179.82 | –1141 | 440 |
| 2022 | BAP | –63.91 | –451 | 517 |
| 2023 | BAP | –78.92 | –445 | 228 |


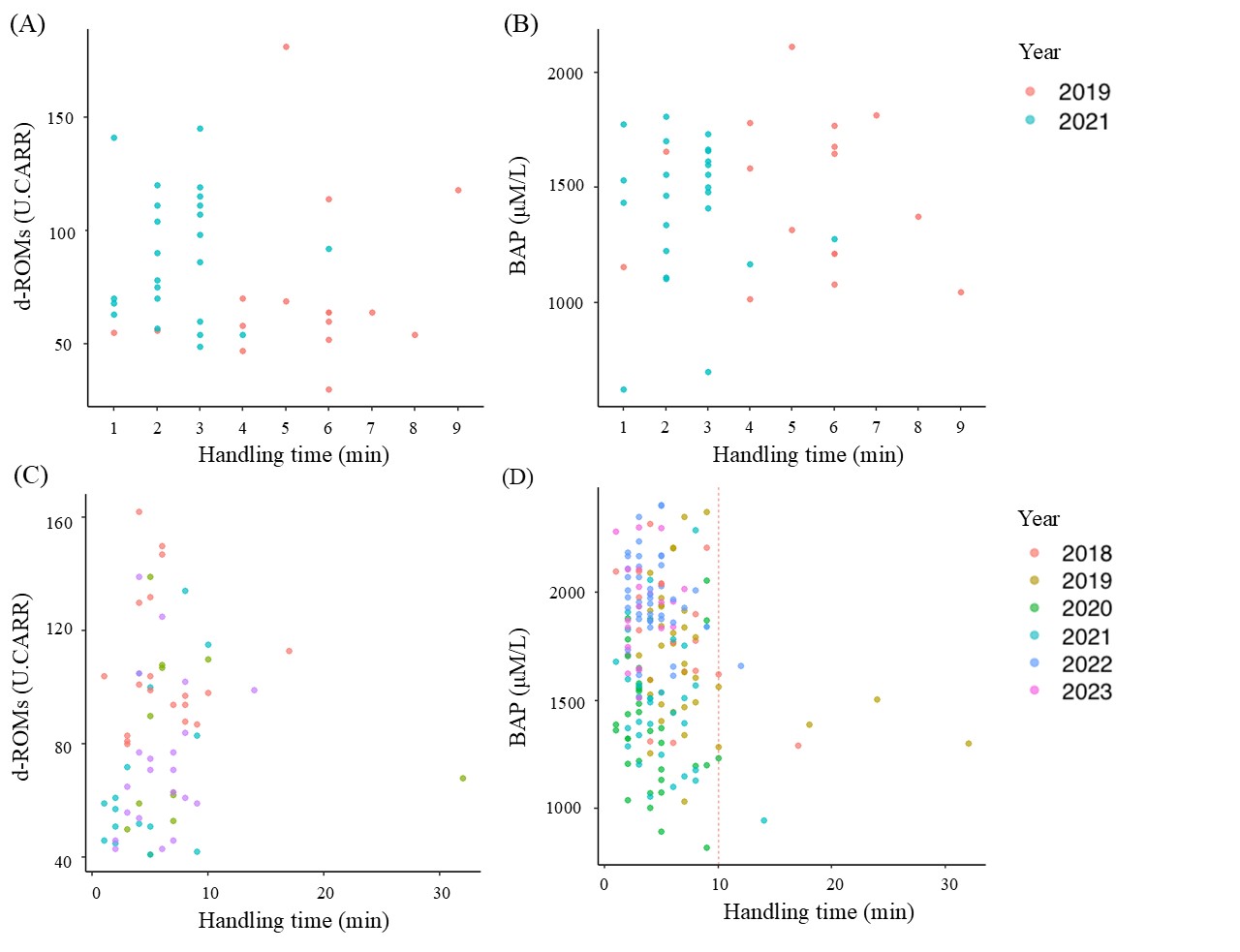


Figure S1. There were no significant relationships between handling time and d-ROMs (A) or BAP (B) in blood samples from black-tailed gulls, nor between handling time and d-ROMs in streaked shearwaters (C). The red vertical line in the BAP figure for streaked shearwaters (D) indicates a handling time of 10 minutes. There were no significant relationships between handling time and BAP in blood samples collected 10 minutes or less from streaked shearwaters.
